# Development of CaReScreen, a Digital Health Tool for Assessing Late Effects and Cancer Rehabilitation Needs in Cancer Survivors: Protocol for a Qualitative Development and Formative Design Study

**DOI:** 10.2196/105443

**Published:** 2026-10-29

**Authors:** Maja Kulosman, Harald Kåre Engan, Unn Merete Fagerli, Kristin Valborg Reinertsen, Kari Sand, Jon Arne Sandmæl, Arne Solberg, Torgrim Tandstad

**Affiliations:** 1 Department of Clinical and Molecular Medicine, Faculty of Medicine and Health Sciences, Norwegian University of Science and Technology, P.O. 8905, Trondheim, Trøndelag, 7491, Norway, 47 72853114; 2 Cancer Clinic, St Olav's University Hospital, Trondheim, Trøndelag, Norway; 3 Unicare, Oslo, Norway; 4 Department of Oncology, Division of Cancer Medicine, Oslo University Hospital, Oslo, Norway; 5 Department of Health Services Research, SINTEF Digital, Trondheim, Trøndelag, Norway; 6 Faculty of Medicine and Health Sciences, Norwegian University of Science and Technology, Trondheim, Trøndelag, Norway

**Keywords:** cancer survivors, cancer rehabilitation, digital health tools, eHealth, need assessment, late effects, digital development, decision support

## Abstract

**Background:**

Due to advances in cancer diagnostics and treatments, life expectancy after cancer and the number of cancer survivors are increasing. Symptoms and late effects frequently occur in cancer survivors, with a negative impact on functioning and participation in society. Digital health tools might be helpful to systematically assess symptom burden of late effects and rehabilitation needs.

**Objective:**

This paper describes the objectives, design, and current status of the Cancer Rehabilitation Screening (CaReScreen) project. The aims are (1) to report the completed development phase of the digital health tool CaReScreen, comprising the exploratory work, the establishment of the interdisciplinary team, the selection of the assessment instrument, and the development of the initial prototype, and (2) to define the objectives, design, and timelines of the ongoing validation of the scoring manual and of the planned large-scale feasibility study.

**Methods:**

Development followed an iterative, qualitative, and participatory design approach guided by established frameworks for digital health development. It was informed by exploratory qualitative work comprising 40 interviews (9 group and 31 individual) involving 72 informants across Norway, which identified systematic assessment of late effects and navigation of rehabilitation services as the primary unmet needs in cancer survivorship follow-up. A 105-item patient-reported outcome measure (European Organization for Research and Treatment of Cancer [EORTC] QLQ-SURV100, supplemented with 5 EORTC QLQ-C30 items) was selected as the main assessment instrument. This paper reports the completed development phase; the ongoing validation study and the planned feasibility study are described in terms of objectives, design, and timelines, but their results are not reported here.

**Results:**

An initial prototype was developed with health care professionals and cancer survivors as intended end users. It consists of a digital report that identifies and graphically visualizes a panoramic view of multidimensional needs, based on a traffic light scoring system: green indicates low symptom burden and high functioning, yellow moderate symptom burden and moderate functioning, and red high symptom burden and low functioning. The project is funded for the period 2022 to 2028. The development phase is completed, as well as early prototype usability and usefulness testing. Validation of the scoring manual is ongoing, with 800 participants included to date and reporting expected in spring 2027. A large-scale feasibility study is planned to commence in 2027.

**Conclusions:**

The CaReScreen interdisciplinary team has developed an initial digital health tool prototype with the main aim of enabling systematic assessment of late effects, functioning, and unmet rehabilitation needs in cancer survivors. The prototype now proceeds to validation and feasibility testing, which will determine its clinical utility and support regulatory approval under the European Medical Device Regulation.

## Introduction

### Background

Due to advances in cancer diagnostics and treatment, the number of people living after a diagnosis of cancer (cancer survivors) is increasing. At present, there are more than 350,000 cancer survivors in Norway [1]. Almost 80% of those diagnosed with cancer are alive 5 years after diagnosis [1]. However, cancer treatment may cause a wide variety of side effects, including late effects that negatively impact quality of life (QoL) [2-4], such as fatigue, cognitive impairments, neuropathy, and insomnia. Late effects may also negatively affect life expectancy and the ability to live an independent life, and data show an increased risk of unemployment in cancer survivors [5,6].

Rehabilitation after cancer treatment aims to help retain independence and improve QoL by increasing functioning and reducing symptoms [7,8], and is shown to be a cost-effective intervention that may reduce the financial burden on society [9]. Thus, international oncology guidelines recommend assessment of rehabilitation needs and interventions to treat both physical and cognitive impairment in cancer survivors [3,7]. However, studies indicate that such services are underutilized [3,7,10,11]. Although more than 60% of cancer survivors report functional impairment and more than 40% report unmet rehabilitation needs, only 2% to 9% are referred to rehabilitation [7,12]. The discrepancy between guideline recommendations and clinical practice may lead to suboptimal follow-up care and support for cancer survivors [13]. During routine oncology follow-up, priority is given to relapse monitoring and treatment of side effects, while rehabilitation needs are less frequently addressed [4,13]. In addition, cancer survivors may lack awareness of the potential benefits of rehabilitation or the availability of such services and supportive care [4,11,14].

The use of patient-reported outcome measures (PROMs) has emerged as an important tool to improve the quality and efficiency of clinical care and follow-up [15]. Studies show that the use of PROMs conserves resources through improved self-management, early recognition of alarming symptoms, and faster action from health care professionals, and contributes to better communication between patients and physicians [16-19].

Several digital health tools have been developed for symptom monitoring in cancer care. However, there is a lack of integrated solutions specifically designed to support systematic identification of late effects in follow-up and referral for rehabilitation needs, while also facilitating coordination across different levels of the health care system.

The Cancer Rehabilitation Screening (CaReScreen) project is part of the Norwegian Pilot Health program funded by the Research Council of Norway. The main objective of CaReScreen is to establish better models for cancer survivorship. The core of CaReScreen is a digital and evidence-based clinical digital health tool that will be developed in this project. It is a novel tool developed to overcome common barriers to clinical implementation by supporting systematic assessment and longitudinal follow-up of late effects and unmet rehabilitation needs in cancer survivors based on PROMs. CaReScreen also aims to facilitate referral and triage to the most efficient rehabilitation level, ranging from complex inpatient rehabilitation to self-directed rehabilitation. The primary end users of the tool are health care professionals and cancer survivors.

This paper describes the planning phase, design, and early development of the CaReScreen digital health tool.

### Objectives

The overall objective of the CaReScreen project is to establish improved models for cancer survivorship care through the development, validation, and implementation of an evidence-based digital health tool. The project comprises several sequential and partly overlapping phases (Figure 1, Table 1). The objectives of the individual phases are distinguished below, and the phases to which they belong are indicated throughout the *Methods* section.

The objectives of the completed development phase, which is the phase reported in this paper, were to (1) conduct exploratory work in order to understand the target problem and identify unmet needs in the follow-up of cancer survivors; (2) establish an interdisciplinary team and a sustainable project structure with defined roles and responsibilities; (3) select an adequate and previously validated assessment instrument; and (4) develop and structure an initial prototype, including a scoring model and a visualization of the resulting report.

**Figure 1.**
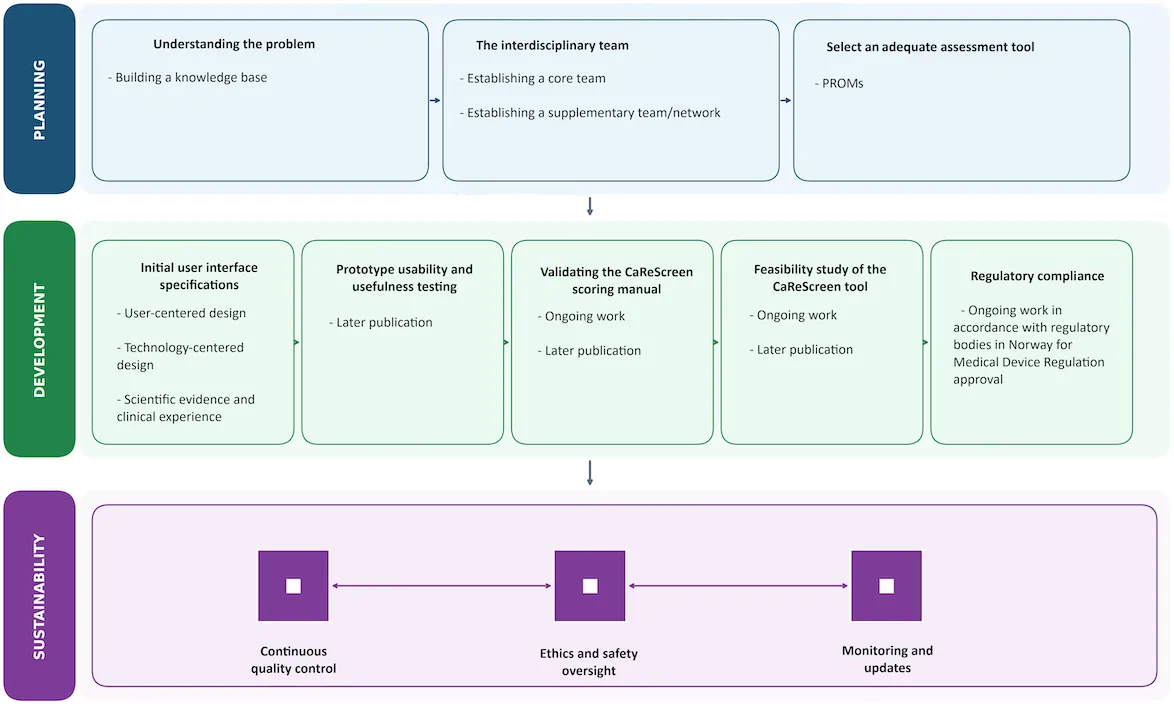
Visualization of the developmental framework of the Cancer Rehabilitation Screening (CaReScreen), including future work and planned publications. This paper describes the planning phase and the initial development of the CaReScreen prototype. PROMs: patient-reported outcome measures.

The objective of the prototype usability and usefulness testing was to explore health care professionals’ perceptions of the usability, clinical usefulness, and potential associated challenges of the CaReScreen prototype.

The objective of the ongoing validation phase is to evaluate whether the proposed threshold values in the CaReScreen scoring manual accurately differentiate between clinically meaningful levels of symptom burden, functioning, and rehabilitation needs.

The objectives of the planned feasibility phase are to evaluate the applicability, usability, and clinical utility of CaReScreen in real-world clinical settings and to generate the clinical evidence required for regulatory approval under the European Medical Device Regulation (MDR).

The results and outcomes of the ongoing and planned phases are outside the scope of this paper and will be reported in separate publications, as summarized in Table 1.

**Table 1.** Overview of the Cancer Rehabilitation Screening development phases, their current status, timelines, and intended reporting^a^.

Phase/activity	Status	Timeline	Reported in
1. Exploratory work (40 interviews, 72 informants; CaReScreen^b^ knowledge base)	Completed	2023	SINTEF report [20]; this paper
2. Establishment of the interdisciplinary team and development plan	Completed (collaboration continues)	From February 2023	This paper
3. Selection of assessment instrument (EORTC^c^ QLQ-SURV100+5 EORTC QLQ-C30 items)	Completed	2023	This paper
4. Development of the initial prototype, scoring model, and report visualization	Completed	2023-2025	This paper
5. Qualitative usability and usefulness testing of the initial prototype with health care professionals	Completed; analysis finished	2025‐2026	Separate manuscript, ready for submission
6. Validation of the CaReScreen scoring manual (n=800)	Ongoing; statistical analysis in progress	Inclusion to be completed in 2026; expected study completion in 2026	Separate publication, spring 2027
7. Large-scale feasibility study in clinical settings	Planned	Commencing in 2027; expected completion in 2028	Separate publication
8. Regulatory activities toward MDR^d^ approval	Ongoing	2025-2028	N/A^e^

a Phases 1 to 4 are reported in this paper.

b CaReScreen: Cancer Rehabilitation Screening.

c EORTC: European Organization for Research and Treatment of Cancer.

d MDR: Medical Device Regulation.

e N/A: not applicable.

## Methods

### Study Overview

The prototype was developed through an iterative, qualitative, and participatory design approach, guided by established frameworks for digital health development (Figure 1). The development process was informed by recommendations for digital health innovation, specifically the framework proposed by Loo et al [21], emphasizing interdisciplinary collaboration, active patient involvement, and iterative design [21]. The framework aims to support the development and implementation of innovative, evidence-based health solutions through close collaboration between research, health care services, and industry. This process implies continuous involvement of end users and interdisciplinary stakeholders across all phases of development, from initial needs assessment through prototype design, to ensure that the tool is grounded in real-world clinical practice. The activities described in the four subsections below have all been completed, and are the activities reported in this paper: (1) conducting the exploratory work to understand the target problem, (2) establishing the interdisciplinary team, (3) selecting an adequate assessment tool, and (4) developing the initial prototype. The subsequent validation, expanded usability, feasibility, and implementation activities are either ongoing or still in planning; their status, timelines, and intended reporting are described in the *Study Status* section and summarized in Table 1, and no results from these activities are presented in this paper.

Reporting follows established guidance for the respective study designs. The exploratory qualitative work is reported in accordance with the COREQ (Consolidated Criteria for Reporting Qualitative Research) [22], a 32-item checklist for interview and focus group studies covering the research team and reflexivity, the study design, and the analysis and findings. COREQ was chosen to make the qualitative basis of the development phase transparent and open to appraisal, as the exploratory work constitutes the primary empirical foundation for the requirement specification and the design of the CaReScreen prototype. The completed checklist is provided as Checklist 1. The ongoing validation of the scoring manual is a cross-sectional study and will be reported in accordance with the STROBE (Strengthening the Reporting of Observational Studies in Epidemiology) statement [23], while reporting of the planned feasibility study will follow the guidance appropriate to its final design.

To ensure meaningful and continuous feedback from the patient perspective, 2 user representatives were involved throughout the planning and early development phases, allowing for direct interaction with the solution in development. Key activities in which user representatives were engaged included group-based discussions and prototype testing.

### Understanding the Problem (Completed)

Broad exploratory work was conducted to identify specific gaps in knowledge and current practice regarding rehabilitation needs, referral processes, rehabilitation services, and decision support tools. In this qualitative process, information was gathered by interviewing cancer survivors and health care professionals. In total, 40 interviews were conducted, comprising 9 group interviews and 31 individual interviews. As the group interviews each included several participants, the 40 interviews involved 72 individual informants in total: 31 informants participated in an individual interview, and 41 informants participated in one of the 9 group interviews. The 72 informants comprised cancer survivors (n=15), health care professionals from the municipal health care services (n=22), health care professionals from the specialist health care services (n=30), health care professionals from private specialized rehabilitation (n=4), and one representative of the voluntary sector (n=1). The findings were compiled into a 351-page report that constitutes the CaReScreen knowledge base [20].

Informants were recruited purposefully through the clinical, municipal, and user organization networks of the project in order to cover the range of stakeholders involved in cancer survivorship follow-up in Norway. The interviews were conducted during 2023 by 11 members of the interdisciplinary team with different occupational backgrounds, in person, by telephone, or by videoconference, using semistructured interview guides covering rehabilitation needs, referral processes, available rehabilitation services, and decision support. The interviews were audio-recorded, transcribed, and analyzed, with interpretations discussed within the research group. A full account of the methods and findings is provided in the SINTEF report [20].

The main findings from the exploratory work were used to establish a problem statement to gain an understanding of the challenges CaReScreen is trying to address. They specifically revealed a need for more comprehensive and systematic assessment of symptoms and late effects and rehabilitation needs following treatment. There is also a significant lack of awareness and overview of available rehabilitation services among both survivors and health care professionals within a fragmented system of public and private providers that is difficult to navigate. This often leads to suboptimal referrals, without considering low-threshold services that could serve as appropriate initial interventions. Additionally, there is a need to reduce inequalities in access to rehabilitation by developing inclusive and user-friendly digital tools that accommodate individuals with diverse needs, including those with lower health literacy, minority backgrounds, or disabilities. In sum, these findings influenced the decisions of the interdisciplinary team for further development of the requirement specification for CaReScreen.

### Establishing the Interdisciplinary Team (Completed)

The CaReScreen interdisciplinary team consists of a core team of researchers, experienced health care professionals working with cancer patients and treatment (doctors, nurses, and physiotherapists), cancer survivors, software developers/engineers, and user interface/user experience designers, as well as private and public Norwegian stakeholders, including members from nonprofit health advocacy groups like the Norwegian Cancer Society. In addition, a supplementary network of collaborators, mainly physicians, psychologists, and statisticians, has been involved in planning and development. The Norwegian digital health company Flow Technologies was selected as the software stakeholder because its operational methods and existing platform aligned closely with the conceptual framework and identified needs of the project.

Before initiation of the CaReScreen project, a development plan was established to ensure a clear and sustainable project structure with defined roles and responsibilities. Due to the iterative nature of the project, CaReScreen has been continuously refined through interdisciplinary collaboration.

### Selecting an Adequate Assessment Tool (Completed)

To assess functioning, late effects, and symptom burden in cancer survivors, several PROMs frequently applied internationally were considered for use in CaReScreen. Eligibility criteria for the questionnaire included prior thorough and systematic validation in European populations and coverage of a comprehensive symptom profile relevant to cancer survivorship in adults. The key requirement was to apply a single questionnaire that systematically captures a wide range of potential symptoms following cancer therapy, rather than using a collection of symptom-specific questionnaires with limited clinical scope.

The questionnaire European Organization for Research and Treatment of Cancer (EORTC) QLQ-SURV100, developed by the EORTC, was selected as the main PROM in CaReScreen [24].

The EORTC QLQ-SURV100 has been designed, developed, and comprehensively tested in the European cancer population. EORTC is currently conducting an international large-scale phase IV trial comprising a psychometric and cross-cultural validation study of the EORTC QLQ-SURV100 [24]. The EORTC QLQ-SURV100 consists of 100 items, including a global health status and QoL scale, functional scales, symptom scales, one symptom checklist, and single items. They are developed to identify levels of symptoms and/or functioning [24,25]. A higher score on the functional scales indicates higher functioning, while a higher score on the symptom scales and single items indicates a greater burden of symptoms [26]. In addition to the 100 items of the EORTC QLQ-SURV100, we included 5 items (items 13‐17) from the EORTC QLQ-C30 in order to obtain a better overview of gastrointestinal complaints [26]. In total, the initial questionnaire included in CaReScreen comprises 105 items. The items in the EORTC questionnaires are scored on a Likert scale ranging from 1 (“not at all”) to 4 (“very much”) [26].

### Initial Development and Structuring of the CaReScreen Prototype (Completed)

A prototype of the digital tool was designed in the Flow Technologies system through an iterative design approach. The development process was further informed by considerations of regulatory alignment with the European MDR, particularly regarding intended purpose, risk classification, and the role of the tool as decision-supportive software.

The first collaborative meeting was held in February 2023, followed by regular interdisciplinary physical and digital meetings. The interdisciplinary research group developed the initial CaReScreen model. Through an iterative process, feedback was continuously elicited from the entire team during the development of the prototype, and changes were initiated accordingly. The collaborative work was mainly digital, since the participants in the interdisciplinary team reside and work across widely dispersed geographic areas of Norway.

### Study Status

The CaReScreen project received funding for the project period 2022 to 2028 and consists of several sequential and partly overlapping developmental phases (Figure 1). The project was formally initiated in late 2022, the exploratory work was carried out from spring to fall 2023, and the first collaborative meeting of the interdisciplinary team was held in February 2023. An overview of all phases, their current status, timelines, and intended reporting is provided in Table 1.

The following phases are completed and are reported in this paper: the exploratory work, the establishment of the interdisciplinary team, the selection of the assessment instrument, and the development of the initial prototype. In addition, initial prototype usability and usefulness testing has been completed. Data collection was performed in February and March 2025, and qualitative analysis for that study was concluded in late 2025. The resulting manuscript is ready for submission; its results are not reported here.

Validation of the CaReScreen scoring manual is currently ongoing. The objective of this study is to evaluate whether the proposed threshold values in the CaReScreen scoring manual accurately differentiate between clinically meaningful levels of symptoms and rehabilitation needs. By July 2026, 800 participants had been included, and the study is expected to be completed in late 2026 and reported in a separate publication in spring 2027.

A large-scale feasibility study in clinical settings is still in planning and is intended to commence in 2027, with expected completion in 2028. The main objective of this study will be to evaluate the applicability of the tool in real-world clinical settings and to further support the regulatory activities related to approval in accordance with the MDR. Feasibility and acceptability will be assessed through recruitment and retention rates, the proportion of participants completing the questionnaire, time to completion, usability, the number and appropriateness of rehabilitation referrals generated through the tool, and qualitative interviews with cancer survivors and health care professionals. Regulatory activities toward MDR approval are ongoing, with expected completion by the end of 2028. No results from the ongoing or planned phases are reported in this paper.

### Statistical Analysis of the Ongoing Validation Study

The development phase reported in this paper followed a qualitative and iterative approach, and no statistical analyses were performed on the material presented here. Statistical methods apply to the ongoing validation of the CaReScreen scoring manual, the results of which will be reported in a separate publication. The planned statistical approach is outlined below.

The validation study is a cross-sectional study. At this time, 800 cancer survivors have been recruited from clinical, municipal, and user organization networks of the project and have completed the CaReScreen questionnaire. A statistician in the interdisciplinary team recommended a target of 600 completed questionnaires, based on a review of relevant literature, to ensure adequate statistical power and data quality. Inclusion has continued beyond this target to compensate for anticipated missing data, and 800 participants had been included by July 2026. Recruitment continues until the statistical analyses are initiated, with an anticipated final sample of approximately 1000 participants.

The outcome under evaluation is the CaReScreen scoring manual. The clinical relevance of the proposed threshold values in the CaReScreen scoring manual will be evaluated against anchor variables from previously validated and clinically relevant questionnaires assessing the most relevant late effects of cancer treatment, which serve as anchors defining clinically meaningful levels of symptom burden, reduced functioning, and rehabilitation need.

Descriptive statistics will be used to characterize the sample and the distribution of scale scores and traffic light categories, and the psychometric properties of the scales will be assessed using established methods. The proposed threshold values will be evaluated against the anchors using established methods for assessing diagnostic accuracy and agreement, and the ability of the thresholds to distinguish between groups expected to differ will be examined. Where applicable, the resulting thresholds will be compared with published EORTC thresholds for clinical importance. The analytic approach will be described in full in the validation publication. Revisions to the scoring manual will be made according to the statistical findings. Where statistical performance and clinical judgment diverge, the final thresholds will be determined by consensus within the interdisciplinary team, and any departure from the statistically optimal cutoff will be reported explicitly, together with its rationale, in the validation publication.

### Ethical Considerations

#### Ethics Approval

The CaReScreen project comprises several distinct studies, each of which has received or will receive a separate ethical assessment. The project in its entirety was reviewed and approved by the Clinic Internal Review Board at the Cancer Clinic, St Olavs Hospital, Trondheim University Hospital, Trondheim, Norway. The Clinic Internal Review Board does not assign formal case numbers to its approvals; approvals are recorded internally at the Cancer Clinic, and documentation can be made available from the corresponding author on request.

The exploratory work reported in this paper was conducted as a needs assessment and service development activity and was assessed to fall outside the scope of the Norwegian Act on Medical and Health Research (the Health Research Act, LOV-2008-06-20-44). Approval from the Regional Committee for Medical and Health Research Ethics (REK) was therefore not required. The protocol and the procedures for processing personal data were assessed by the Data Protection Services for Research (reference number 218300).

The qualitative study of the usability and usefulness of the early prototype was likewise assessed to fall outside the scope of the Norwegian Health Research Act, and ethics approval was therefore not sought from REK. The study protocol and the procedures involving personal data were approved by the Clinic Internal Review Board at the Cancer Clinic, St Olavs Hospital, Trondheim, Norway.

The ongoing validation study of the CaReScreen scoring manual was assessed by the REK and was determined not to require formal ethical approval (reference number 864518), as it falls outside the scope of the Norwegian Health Research Act. The protocol and the procedures for processing personal data were approved by the Clinic Internal Review Board and through a data protection impact assessment at St Olavs Hospital.

For the large-scale feasibility study that is currently being planned, approval will be sought from the Regional Committee for Clinical Trials of Pharmaceuticals and Medical Devices before the study is initiated.

#### Informed Consent

For all the studies described above, all participants provided or will provide written informed consent prior to participation. Participation is voluntary, and participants are informed of their right to withdraw their consent at any time and without any consequences for their treatment or follow-up.

#### Privacy and Confidentiality

All data collected in the CaReScreen studies are deidentified or pseudonymized prior to analysis and are stored on secure research servers in accordance with the General Data Protection Regulation and applicable Norwegian legislation. No individually identifiable participant information is presented in this manuscript or in any of the accompanying figures.

#### Compensation

Participants in the studies described in this paper did not receive any financial compensation for their participation.

## Results

### Overarching Specification

Based on the findings from the exploratory work and feedback and criteria established by the interdisciplinary team, an initial requirement specification was established, defining the core functions, users, and goals of CaReScreen. The overarching specification was to develop a user- and technology-centered digital health tool based on scientific evidence and clinical experience to ensure usability while maintaining its scientific foundation and usefulness.

### Visualization and Interpretation of the Questionnaire Report

To ensure user-friendliness, the interdisciplinary team concluded that a scoring system was necessary to display the levels of the different symptoms and functioning. Inspired by other digital health tools, such as the web-based dialogue and decision support tool Ambuflex, a digital report based on a traffic light model (green, yellow, red) was developed [19]. Based on the original scores on the Likert scale ranging from 1 to 4 in the original EORTC QLQ-SURV100 questionnaire, the sum scores for each function, symptom, and symptom category were transformed into 3 groups (green, yellow, red), based on the levels of symptoms and functioning. In our model, green indicates a low burden of symptoms/high functioning, yellow a moderate burden of symptoms/moderate functioning, and red a high burden of symptoms/low functioning. In addition, safeguards were incorporated into the scoring system to prevent single responses that deviate from the average response pattern from being overlooked, using a visual alert indicator. To ensure the robustness of the scoring system and the visual approach, a validation study is currently being conducted to establish its clinical relevance.

In clinical use, CaReScreen is completed by the cancer survivor as a web-based questionnaire in advance of a follow-up consultation. The generated report is reviewed together with the health care professional during the consultation and is intended to support decisions on referral to rehabilitation services.

## Discussion

### Principal Findings

The findings from the CaReScreen exploratory work highlight a fragmented follow-up and rehabilitation landscape and limited service visibility. Other studies also show that cancer survivors have fragmented care pathways and survivorship follow-up [27], and that rehabilitation services are insufficiently integrated and coordinated in oncological care, often resulting in underutilization and delayed or inappropriate referrals [3,28].

Health care professionals in Norway lack appropriate tools to structure and acknowledge the multifaceted symptom profiles often experienced by cancer survivors. Limited knowledge is a key barrier to cancer rehabilitation, including uncertainty about available services, restricted access, and inadequate identification and documentation of functional impairments by physicians [28-31]. Digital health tools to support structured symptom assessment and transparent overviews of rehabilitation services could potentially mitigate some of these challenges. These key challenges formed the foundation for the development and design of CaReScreen, which aims to address and optimize them.

There are some challenges from the exploratory work that remain insufficiently addressed. Currently, no national overview of rehabilitation services in Norway exists. The services are provided at multiple levels (including low-threshold services and voluntary organizations), and maintaining such an overview would require resources and a clear allocation of responsibility that are not currently in place. This is also highlighted in other studies as a barrier to rehabilitation [29].

Possible inequalities in access to rehabilitation services were also revealed, particularly affecting individuals with low education, limited health literacy, and minority groups. Digital health tools may fail to accommodate the technical needs required to support the low digital literacy commonly observed in these populations [32,33]. In the process of digital development, such requirements may be insufficiently prioritized due to time and funding constraints or because of limited expertise in inclusive technology design [32,33].

It is uncertain whether a digital health tool is sufficient to address such complex social system–level problems. However, we propose that adopting the user-centered design approach may increase the usability and uptake of CaReScreen among marginalized populations.

### Strengths of the Applied Approach

A key strength of our development processes was the involvement of a carefully selected, diverse interdisciplinary team. Throughout the CaReScreen development process, priority was given to integrating expertise from all stakeholders involved in cancer survivorship follow-up in Norway, with the aim of improving existing rehabilitation services. This interdisciplinary and inclusive approach is further reflected in our selected design methodologies to ensure alignment with end-user needs and real-world contexts [34].

CaReScreen is envisioned to be available to European cancer survivors and health care professionals, including primary, specialist, and rehabilitation services, nurses, physicians, and physiotherapists involved in cancer treatment and follow-up. End-user involvement was central throughout the development phase of CaReScreen. User preferences, expectations, and perspectives were systematically incorporated across the entire process, from initial conceptualization to the development of the initial prototype. The user-centered approach was applied to ensure that the needs, preferences, and real-world contexts of the intended end users shaped both the structure and functionality of CaReScreen [35,36]. This was done to achieve an intuitive solution and to account for varying digital skills among end users [34].

This design philosophy is intended to support iterative and adaptive development throughout the lifecycle of CaReScreen, ensuring that continuous user feedback informs and drives further digital refinement [34]. CaReScreen may represent a solution to improve and establish more effective care, follow-up, and rehabilitation while addressing the needs and demands of patients and health care professionals. To use the full capabilities of the digital platform developed by Flow Technologies while ensuring technical robustness, reliability, and alignment with current technological standards, it was important to integrate technology- and user-centered design principles [21].

Finally, grounding CaReScreen in evidence-based science and clinical experience will ensure scientifically valid content and recommendations and applicability in complex health care settings in alignment with established best practices. The single, comprehensive PROM was deliberately selected to balance clinical relevance with feasibility in follow-up care. Given the complexity and heterogeneity of survivorship-related symptoms, emphasis was placed on selecting a PROM that had undergone thorough and systematic validation in European populations and provided broad coverage of symptoms and functioning relevant to cancer survivorship [24]. While several validated PROMs were available, the use of multiple symptom-specific instruments may increase respondent burden and limit clinical usability. From this perspective, we consider the composite questionnaire, consisting of the EORTC QLQ-SURV100 with 5 added questions from the EORTC QLQ-C30, well suited to support systematic symptom assessment within CaReScreen.

### Limitations

At the current stage of development, CaReScreen has some important limitations. It remains an early-stage prototype and requires further rigorous testing before it can be considered for use in clinical practice. Although usability and usefulness testing of the initial prototype has been conducted among health care end users, additional evaluation is necessary to ensure robustness, validity, and broader applicability.

To address these limitations, a validation study of the CaReScreen scoring manual is currently ongoing, and a large-scale feasibility study is being planned by the CaReScreen project members to evaluate the applicability of the tool in real-world clinical settings. The design, status, and expected completion of both studies are described in the *Study Status* section and in Table 1. Through these studies, we aim to develop a mature digital health tool intended to enhance cancer survivorship care, particularly with regard to follow-up and referral to rehabilitation services.

Integration into existing electronic health record systems may constitute a significant barrier to implementation and requires regulatory approval. Processes to meet regulatory requirements through engagement with Norwegian regulatory authorities for MDR approval are currently in progress.

### Conclusion

The CaReScreen interdisciplinary team has developed an initial prototype of a digital health tool aiming to enable systematic assessment of late effects, functioning, and rehabilitation needs during (longitudinal) follow-up of cancer survivors. To the best of our knowledge, no comparable tools are currently available.

Having a robust initial prototype should help CaReScreen move forward to overcome common challenges associated with the implementation of such tools in clinical practice. If validated and successfully implemented, CaReScreen has the potential to support more systematic referral to rehabilitation services across Norway. By closing some well-documented gaps between clinical guidelines and current practice regarding rehabilitation needs, CaReScreen might contribute to improved cancer survivorship care and, ultimately, QoL among survivors.

## Supplementary material

10.2196/105443 Multimedia Appendix 1 Draft of Figure 1.

10.2196/105443 Checklist 1 COREQ checklist.

10.2196/105443 Peer Review Report 1 Report from funding agency in Norwegian.

## Abbreviations

CaReScreen: Cancer Rehabilitation Screening
COREQ: Consolidated Criteria for Reporting Qualitative Research
EORTC: European Organization for Research and Treatment of Cancer
MDR: Medical Device Regulation
PROM: patient-reported outcome measure
QoL: quality of life
REK: Regional Committee for Medical and Health Research Ethics

## References

[1] (2025). Cancer in Norway 2025 - Cancer incidence, mortality, survival and prevalence in Norway. https://www.fhi.no/en/publ/2026/cancer-in-norway-2025/.

[2] Wéber A, Laversanne M, Nagy P, Kenessey I, Soerjomataram I, Bray F (2023). Gains in life expectancy from decreasing cardiovascular disease and cancer mortality - an analysis of 28 European countries 1995-2019. Eur J Epidemiol.

[3] Kline-Quiroz C, Andrews C, Martone P (2024). Rehabilitation in oncology care guidelines: a gap analysis. J Natl Compr Canc Netw.

[4] Hussey C, Hanbridge M, Dowling M, Gupta A (2024). Cancer survivorship: understanding the patients’ journey and perspectives on post-treatment needs. BMC Sports Sci Med Rehabil.

[5] de Boer AG, Torp S, Popa A (2020). Long-term work retention after treatment for cancer: a systematic review and meta-analysis. J Cancer Surviv.

[6] Kemp E, Ugalde A, Marshall S (2025). Factors associated with employment outcomes in cancer survivors: a systematic scoping review spanning a decade of international research. Support Care Cancer.

[7] Stout NL, Santa Mina D, Lyons KD, Robb K, Silver JK (2021). A systematic review of rehabilitation and exercise recommendations in oncology guidelines. CA Cancer J Clin.

[8] Miller KD, Nogueira L, Mariotto AB (2019). Cancer treatment and survivorship statistics, 2019. CA Cancer J Clin.

[9] Silver JK, Baima J, Mayer RS (2013). Impairment-driven cancer rehabilitation: an essential component of quality care and survivorship. CA Cancer J Clin.

[10] Pergolotti M, Deal AM, Lavery J, Reeve BB, Muss HB (2015). The prevalence of potentially modifiable functional deficits and the subsequent use of occupational and physical therapy by older adults with cancer. J Geriatr Oncol.

[11] Stubblefield MD (2017). The underutilization of rehabilitation to treat physical impairments in breast cancer survivors. PM R.

[12] Thorsen L, Gjerset GM, Loge JH (2011). Cancer patients’ needs for rehabilitation services. Acta Oncol.

[13] Silver JK (2017). Integrating rehabilitation into the cancer care continuum. PM R.

[14] McEwen S, Rodriguez AM, Martino R (2016). “I didn’t actually know there was such a thing as rehab”: survivor, family, and clinician perceptions of rehabilitation following treatment for head and neck cancer. Support Care Cancer.

[15] Yang LY, Manhas DS, Howard AF, Olson RA (2018). Patient-reported outcome use in oncology: a systematic review of the impact on patient-clinician communication. Support Care Cancer.

[16] Bennett AV, Jensen RE, Basch E (2012). Electronic patient-reported outcome systems in oncology clinical practice. CA Cancer J Clin.

[17] Johansen MA, Henriksen E, Horsch A, Schuster T, Berntsen GKR (2012). Electronic symptom reporting between patient and provider for improved health care service quality: a systematic review of randomized controlled trials. Part 1: state of the art. J Med Internet Res.

[18] Snyder CF, Jensen R, Courtin SO, Wu AW, Website for Outpatient QOL Assessment Research Network (2009). PatientViewpoint: a website for patient-reported outcomes assessment. Qual Life Res.

[19] Schougaard LMV, Larsen LP, Jessen A (2016). AmbuFlex: tele-patient-reported outcomes (telePRO) as the basis for follow-up in chronic and malignant diseases. Qual Life Res.

[20] Torberntsson S, Hansen NV, Sand K, Landstad I (2024). Vitenskapelig rapport. Innsiktsfase i CaReScreen [Report in Norwegian]. https://www.ntnu.no/documents/10275/1402558767/CaReScreen_Rapport+fra+innsiktsarbeidet_medium.pdf/9f9cd5cb-7baa-101b-68f7-9e5841b5f92a?t=1792743031735.

[21] Loo RTJ, Nasta F, Macchi M (2025). Recommendations for successful development and implementation of digital health technology tools. J Med Internet Res.

[22] Tong A, Sainsbury P, Craig J (2007). Consolidated Criteria for Reporting Qualitative Research (COREQ): a 32-item checklist for interviews and focus groups. Int J Qual Health Care.

[23] von Elm E, Altman DG, Egger M (2007). Strengthening the Reporting of Observational Studies in Epidemiology (STROBE) statement: guidelines for reporting observational studies. BMJ.

[24] van Leeuwen M, Kieffer JM, Young TE (2023). Phase III study of the European Organisation for Research and Treatment of Cancer Quality of Life cancer survivorship core questionnaire. J Cancer Surviv.

[25] Tolstrup LK, Dieperink KB, Van Leeuwen M (2024). Health-related quality of life in Danish cancer survivors referred to a late effects clinic: a prospective cohort study. Acta Oncol.

[26] Aaronson NK, Ahmedzai S, Bergman B (1993). The European Organization for Research and Treatment of Cancer QLQ-C30: a quality-of-life instrument for use in international clinical trials in oncology. J Natl Cancer Inst.

[27] Pinheiro LC, Reshetnyak E, Safford MM, Nanus D, Kern LM (2020). Differences in ambulatory care fragmentation between cancer survivors and noncancer controls. Cancer.

[28] Navntoft S, Andreasen J, Petersen KS, Rossau HK, Jørgensen L (2024). Barriers and facilitators to cancer rehabilitation for patients with head and neck or lung cancer-a scoping review mapping structural and healthcare professionals’ perspectives. Disabil Rehabil.

[29] Pergolotti M, Alfano CM, Cernich AN (2019). A health services research agenda to fully integrate cancer rehabilitation into oncology care. Cancer.

[30] Maiwald P, Weis J, Kurlemann U (2022). Barriers to utilisation of cancer rehabilitation from the expert’s view: a cross-sectional survey. Eur J Cancer Care (Engl).

[31] Hodyl N, Mason G, Ribbons K (2024). Barriers and enablers for accessing rehabilitation services: findings from the Rehabilitation Choices Study, part 1-healthcare professionals’ perspectives. Health Expect.

[32] Western MJ, Smit ES, Gültzow T (2025). Bridging the digital health divide: a narrative review of the causes, implications, and solutions for digital health inequalities. Health Psychol Behav Med.

[33] Lyles CR, Nguyen OK, Khoong EC, Aguilera A, Sarkar U (2023). Multilevel determinants of digital health equity: a literature synthesis to advance the field. Annu Rev Public Health.

[34] Tzimourta KD (2025). Human-centered design and development in digital health: approaches, challenges, and emerging trends. Cureus.

[35] Dabbs ADV, Myers BA, Mc Curry KR (2009). User-centered design and interactive health technologies for patients. Comput Inform Nurs.

[36] van Gemert-Pijnen JEWC, Nijland N, van Limburg M (2011). A holistic framework to improve the uptake and impact of eHealth technologies. J Med Internet Res.

