# Supplementary material for: Development of CaReScreen, a Digital Health Tool for Assessing Late Effects and Cancer Rehabilitation Needs in Cancer Survivors: Protocol for a Qualitative Development and Formative Design Study
Source: JMIR Res Protoc. 2026 Oct 29;15:e105443. doi: 10.2196/105443 (PMC13718741; doi:10.2196/105443)
Supplement: Supplementary file 1 [file resprot-v15-e105443-s003.pdf]

Prosjektvurdering – Karaktersammendrag og administrasjonens vurdering av relevans

Prosjektnummer: 331870
Prosjekttittel: CaReScreen: A clinical decision support system for cancer rehabilitation

	Vurderingskriterium	Karakter
1	**Forskning og innovasjon**	**6**
2	**Virkninger og effekter**	**5**
3	**Gjennomføring**	**6**
4	**Relevans for utlysningen**<br>*Søknaden svarer svært godt på kriteriet. Den har svært få, uvesentlige, mangler.*<br><br>*Søknaden tilfredsstiller i stor grad utlysningens krav og forventinger til prosjektansvarlig og til samarbeidskonstellasjon.*<br><br>*Søknaden tilfredsstiller i stor grad forventinger til og kjennetegn ved et Pilot Helse prosjekt som beskrevet i utlysningen. Det fremstår i stor grad sannsynlig at prosjektet vil kunne lede til faktisk implementering og skalering av innovasjonen.*<br><br>*Prosjektet synes i stor grad å samsvare med de tematiske prioriteringene i utlysningen.*	**6**
	**Hovedkarakter***	**6**

Karakterskala: 7, 6, 5, 4, 3, 2, 1 (7 er best)

*) Hovedkarakter er et uttrykk for hvor godt prosjektet oppfyller intensjoner og formål for søknadstypen med utgangspunkt i bedømmelsen av de ulike kriteriene. Hovedkarakter 3 eller lavere forteller at prosjektet har en avgjørende svakhet i form av karakter 3, 2, 1 på minst ett av kriteriene vurdert av panelet.

Karakterene er en del av underlaget ved det besluttende organs behandling av søknaden.

Forskningsrådet

Vurdering av søknad sendt til Norges forskningsråd

Søknad

Prosjektnummer	331870
Prosjekttittel	CaReScreen: A clinical decision support system for cancer rehabilitation
Prosjektleder	Engan, Harald
Prosjektansvarlig	Unicare AS
Søknadstype/variant	Innovasjonsprosjekt / Pilot
Tema	Helse-, omsorgs- og velferdstjenester
Program/Aktivitet	Helseinnovasjon
Saksbehandler	Ina Kathrine Dahlsveen

Bekreftelse

Ved å levere dette skjema i utfylt stand, bekrefter jeg / vi følgende forhold (dette gjelder for den enkelte ekspert eller panelet):

- Jeg/vi er habil til å vurdere denne søknaden. Se Forskningsrådets bestemmelser om habilitet og tillit.	Ja
- Jeg/vi har lest og gjort meg/oss kjent med de kriteriene jeg/vi er bedt om å vurdere søknaden etter og retningslinjene for bruk av karakterskalaen. Karakterskalaen skal brukes for å reflektere absolutte verdier, og karakterer skal ikke settes relativt til andre søknader som panelet/fageksperten vurderer.	Ja
- Jeg/vi er kjent med og har akseptert bestemmelsene for vurdering av søknader for Norges Forskningsråd. Se Bestemmelser for eksperter / panel som skal vurdere søknader for Norges forskningsråd.	Ja
- Jeg/vi er kompetent(e) til å foreta denne vurderingen.	Ja

Karakteroversikt

Kriterier	Karakter
Forskning og innovasjon | Pilot-H	6
Virkninger og effekter | Pilot-H	5
Gjennomføring | Pilot-H	6

Kriterier

Forskning og innovasjon | Pilot-H

.

I hvilken grad representerer prosjektet en ambisiøs innovasjon som understøttes av relevante FoU-aktiviteter av god kvalitet?

• I hvilken grad representerer innovasjonen noe nytt?
• I hvilken grad vil innovasjonen bidra til å løse en utfordring og/eller møte et erkjent behov i helsetjenesten?
• I hvilken grad retter innovasjonen seg mot nye markedsmuligheter for bedriftene som er med i prosjektet?

• I hvilken grad bygger prosjektet på relevant og oppdatert kunnskap?
• I hvilken grad benytter prosjektet relevante og anerkjente FoU-metoder?
• I hvilken grad er FoU-aktivitetene nødvendige for å lykkes med innovasjonen?

Innovasjonen synes i meget stor grad å representere noe nytt og ambisiøst i form av et økosystem for rehabilitering av kreftpasienter som inkluderer både nye tjenestemodeller, beslutningsverktøy og digitale rehabiliteringstilbud.

Behovet for innovasjonen synes i stor grad være forankret i helsetjenesten og spesielt hos pasientene. Det vurderes at innovasjonen(e) som det tas sikte på å utvikle i prosjektet i stor grad vil møte behovet.

Innovasjonen synes i stor grad å kunne gi nye markedsmuligheter for bedriftene i prosjektet.

Det fremstår fra søknaden at prosjektet i stor grad bygger på relevant og oppdatert kunnskap.

FoU-metodene i prosjektet synes å være meget relevante og anerkjente. Det kunne ha væt mer tydelig hvilken rolle utviklingen av digitale rehabiliteringsmetoder har og hvordan det skal foregå.

FoU-aktivitetene synes i stor/meget stor grad å være nødvendige for å lykkes med innovasjonen.

Valgt karakter: 6 - Svært godt
Søknaden svarer svært godt på kriteriet. Den har svært få, uvesentlige, mangler.

Virkninger og effekter | Pilot-H

.

I hvilken grad legger prosjektet til rette for gevinster og verdiskaping både i de offentlige virksomhetene og i norsk næringsliv, og også grunnlag for andre positive samfunnsmessige effekter? I hvilken grad er de potensielle virkningene og effektene av prosjektet tydelig formulert og troverdige?

• I hvilken grad vil prosjektet ha potensial for bærekraftig verdiskaping i norsk næringsliv i form av bedriftsøkonomiske gevinster?
• I hvilken grad vil prosjektet gi grunnlag for gevinster og verdiskaping i helsesektoren i form av en mer bærekraftig helse- og omsorgstjeneste?
• I hvilken grad kan prosjektet gi andre positive eksterne effekter for pasienter, innbyggere og samfunn eller i form av kunnskapsspredning?

• I hvilken grad gir planen for implementering av FoU-resultatene og realisering av gevinster grunnlag for at prosjektet kan oppnå de virkningene og effektene som er beskrevet? Er planen relevant og dekkende, for eksempel når det gjelder spørsmål om
- behov og planer for tilpasning av organisasjonen, systemer og/eller arbeidsprosesser,
- behov og planer for investeringer og allokering av ressurser,
- håndtering av IPR og regulatoriske godkjenninger,
- utvikling av forretningsmodeller og/eller planer for kommersialisering inkl. partnerskap,
- risikovurderinger mht. for eksempel implementering og/eller markedsadgang,
- formidling og kommunikasjon

Prosjektet synes i meget stor grad å kunne legge til rette for bedriftsøkonomiske gevinster og bærekraftig verdiskaping i norsk næringsliv, men potensialet kunne ha vært bedre sannsynliggjort spesielt mht offentlig betalingsvilje, fordeling av kostnader og skalering av teknologien.

Søknaden beskriver et betydelig potensial for gevinster og verdiskaping i helsesektoren, men potensialet kunne ha vært tydeligere formulert og underbygget mht. samfunnsøkonomisk nytte og bærekraft.

Prosjektet synes å kunne legge til rette for andre positive eksterne effekter i form av bedre samhandling mellom tjenestenivå, kunnskapsutvikling og kunnskapsspredning. Hvis prosjektet lykkes, vil det særlig kunne få meget store positive effekter for pasienter og pårørende.

Planen for implementering av FoU-resultatene fremstår som et godt grunnlag for å kunne realisere gevinstene og verdiskapingen som er beskrevet.
Forretningsplan og kommersialiseringsstrategi kunne ha vært bedre beskrevet.
Behovet for tilpasning av organisasjon, systemer eller arbeidsprosesser kunne ha være bede adressert.

Valgt karakter: 5 - Meget godt
Søknaden svarer meget godt på kriteriet, men den har noen få mangler.

Gjennomføring | Pilot-H

.

I hvilken grad gir prosjektplanen og prosjektgruppen et godt grunnlag for å få gjennomført FoU-aktivitetene, og for å få nyttiggjort resultatene?

• I hvilken grad har FoU-prosjektplanen solide og gjennomarbeidede mål, arbeidspakker, milepæler, ressursinnsats og relevante risikovurderinger?
• I hvilken grad er planer for brukermedvirkning relevant og dekkende?

• I hvilken grad sikrer prosjektgruppen den nødvendige kompetansen og ekspertisen for å gjennomføre FoU-prosjektet på en effektiv måte med tilstrekkelig kapasitet?
• I hvilken grad fremtrer prosjektet som strategisk forankret hos partnerne med en med en tydelig og relevant fordeling av budsjett, roller og ansvar?
• I hvilken grad har prosjektleder relevant kompetanse og erfaring for å lede et FoU-prosjekt rettet mot innovasjon og verdiskaping med offentlig-privat samarbeid?

• I hvilken grad har prosjektgruppen den nødvendige kompetansen og kapasiteten til å sikre at FoU-resultatene blir nyttiggjort og gevinster realisert?

FoU-prosjektplanen fremstår som et meget godt utgangpunkt for å få gjennomført FoU-aktivitetene.

Det fremstår ut ifra søknaden at prosjektet i stor grad har tilgang på nødvendig kompetanse og ekspertise for å gjennomføre prosjektet.

Prosjektet fremstår godt forankret hos partnere, men noe skjevfordeling mellom offentlige og private partnere. Rolle- og ansvarfordelingen mellom partnerne fremstår godt tilpasset prosjektet.

Prosjektleder ser ut til å ha gode forutsetninger til å lede prosjektet.

Prosjektgruppen ser i middels stor grad ut til å inneha nødvendige kompetansen og kapasiteten til å sikre at FoU-resultatene blir nyttiggjort og gevinster realisert.

Valgt karakter:	6 - Svært godt<br>Søknaden svarer svært godt på kriteriet. Den har svært få, uvesentlige, mangler.

Sjekkpunkter

Kommentar til sjekkpunkter
