# Supplementary material for: Development of CaReScreen, a Digital Health Tool for Assessing Late Effects and Cancer Rehabilitation Needs in Cancer Survivors: Protocol for a Qualitative Development and Formative Design Study
Source: JMIR Res Protoc. 2026 Oct 29;15:e105443. doi: 10.2196/105443 (PMC13718741; doi:10.2196/105443)
Supplement: Supplementary file 2 [file resprot-v15-e105443-s001.docx]

| **Planning** | **Understanding the problem**   - Building the knowledge base | **Find the interdisciplinary team**   - *Core team:*   Patient representatives  Health care professionals  Physicians  Physiotherapists  Nurses  Researchers  Project managers  Software developers/engineers  Stakeholder Organizations   - *Supplementary team:*   Psychologists  Physicians  Statisticians | **Select the adequate Assessment tool**     - PROMS |  |  |
| --- | --- | --- | --- | --- | --- |
| **Development** | **Initial user interface specifications**   - User‑centered design - Technology‑centered design - Scientific evidence and clinical experience | **Prototype Usability and Usefulness tests**  (Later publication) | **Validation of the CaReScreen Scoring manual**  (Ongoing work and later publication) | **Feasibility Study of the CaReScreen tool**  (Ongoing Work and later publication) | **Regulatory compliance**  (Ongoing Work with regulatory bodies in Norway for Medical Device Regulation approval) |
| **Sustainability** | **Continuous quality control, in addition ethics and safety oversight** |  |  |  |  |
