# Supplementary material for: Development of CaReScreen, a Digital Health Tool for Assessing Late Effects and Cancer Rehabilitation Needs in Cancer Survivors: Protocol for a Qualitative Development and Formative Design Study
Source: JMIR Res Protoc. 2026 Oct 29;15:e105443. doi: 10.2196/105443 (PMC13718741; doi:10.2196/105443)
Supplement: Supplementary file 3 [file resprot-v15-e105443-s002.docx]

**Appendix: COREQ (COnsolidated criteria for REporting Qualitative research) checklist**

Manuscript #105443 — Development of CaReScreen, a Digital Health Tool for Assessing Late Effects and Cancer Rehabilitation Needs in Cancer Survivors: Protocol for a Qualitative Development and Formative Design Study. JMIR Research Protocols.

*The checklist refers to the exploratory qualitative work reported in this protocol (40 interviews, 72 informants, 2023), which is reported in full in the SINTEF report cited as reference 23.*

| **No.** | **Item** | **Guide question / description** | **Reported in** | **Author response** |
| --- | --- | --- | --- | --- |
| **Domain 1: Research team and reflexivity — Personal characteristics** | | | | |
| 1 | Interviewer / facilitator | Which author/s conducted the interview or focus group? | Methods, Understanding the Problem | Interviews and focus groups were conducted by a multidisciplinary team of eleven researchers, innovation advisors and master students from SINTEF, Unicare (provider of occupational health services and a research institution), The Centre for Health Innovation (health hub for interaction, research, innovation and welfare technology solution), St. Olavs hospital, Trondheim University hospital, Nord University and Oslo Metropolitan University. Different team members conducted different interviews depending on practical considerations and availability. |
| 2 | Credentials | What were the researcher's credentials? e.g. PhD, MD | Not reported | The interview team included master's students as well as researchers with master's and PhD degrees in health sciences, social sciences, technology and related fields. |
| 3 | Occupation | What was their occupation at the time of the study? | Not reported | The interview team included senior researchers, researchers, advisors, innovation specialists, healthcare professionals and master's students involved in healthcare research and service development. |
| 4 | Gender | Was the researcher male or female? | Not reported | The interview team consisted of eight female and three male researchers. |
| 5 | Experience and training | What experience or training did the researcher have? | Not reported | The interviewers had experience with qualitative methods, user-centred innovation processes and stakeholder interviews in health and care services. Several had formal training in qualitative research methods. |
| **Domain 1: Research team and reflexivity — Relationship with participants** | | | | |
| 6 | Relationship established | Was a relationship established prior to study commencement? | Not reported | The interviewers had no prior relationship with informants. |
| 7 | Participant knowledge of the interviewer | What did the participants know about the researcher? | Not reported | Informants received written information about the CaReScreen project and its purpose and provided written informed consent prior to participation. No information was disclosed about the interviewers themselves. |
| 8 | Interviewer characteristics | What characteristics were reported about the interviewer/facilitator? e.g. bias, assumptions, reasons and interests in the research topic. | Not reported | No characteristics were reported about the interviewers, except some information about occupational background, e.g. if they are a healthcare professional or not. |
| **Domain 2: Study design — Theoretical framework** | | | | |
| 9 | Methodological orientation and theory | What methodological orientation was stated to underpin the study? | Methods, SINTEF report (23) | The manuscript and the SINTEF report state that the interviews were 'analyzed'. But a qualitative content analysis was performed. |
| **Domain 2: Study design — Participant selection** | | | | |
| 10 | Sampling | How were participants selected? | Methods, Understanding the Problem, SINTEF report (23) | Purposive sampling, to cover the range of stakeholders involved in cancer survivorship follow-up in Norway. |
| 11 | Method of approach | How were participants approached? | SINTEF report (23) | Informants were recruited through the clinical, municipal and user organization networks of the project. Mode of first contact of the cancer survivors were done by professionals not involved in the project. |
| 12 | Sample size | How many participants were in the study? | Methods, Understanding the Problem, SINTEF report (23) | 72 informants took part in 40 interviews (9 group interviews with 41 informants, and 31 individual interviews). |
| 13 | Non-participation | How many people refused to participate or dropped out? Reasons? | Not reported | Not recorded. |
| **Domain 2: Study design — Setting** | | | | |
| 14 | Setting of data collection | Where were the data collected? | Methods, Understanding the Problem, SINTEF report (23) | Interviews were conducted in person, by telephone or by video conference. |
| 15 | Presence of non-participants | Was anyone else present besides the participants and researchers? | Not reported | No |
| 16 | Description of sample | What are the important characteristics of the sample? | Methods, Understanding the Problem, SINTEF report (23) | Cancer survivors (n=15), health care professionals from municipal health care services (n=22), from specialist health care services (n=30), from private specialized rehabilitation (n=4), and one representative of the voluntary sector (n=1). Interviews were conducted during 2023. |
| **Domain 2: Study design — Data collection** | | | | |
| 17 | Interview guide | Were questions, prompts, guides provided by the authors? Was it pilot tested? | Methods, Understanding the Problem; SINTEF report (23) | Semi-structured interview guides covered rehabilitation needs, referral processes, available rehabilitation services and decision support. The guides are documented in the SINTEF report (23). The guides were not pilot tested. |
| 18 | Repeat interviews | Were repeat interviews carried out? If yes, how many? | SINTEF report (23) | No repeat interviews were carried out. |
| 19 | Audio / visual recording | Did the research use audio or visual recording to collect the data? | Methods, Understanding the Problem, SINTEF report (23) | Yes. Interviews were audio recorded and transcribed. |
| 20 | Field notes | Were field notes made during and/or after the interview or focus group? | Not reported | No. |
| 21 | Duration | What was the duration of the interviews or focus group? | SINTEF report (23) | The mean interview duration was 51 minutes and 6 seconds. |
| 22 | Data saturation | Was data saturation discussed? | Methods, SINTEF report (23) | Recruitment aimed at breadth of stakeholder perspectives rather than saturation. |
| 23 | Transcripts returned | Were transcripts returned to participants for comment and/or correction? | SINTEF report (23) | The transcripts were not returned to the participants for comment and/or correction. |
| **Domain 3: Analysis and findings — Data analysis** | | | | |
| 24 | Number of data coders | How many data coders coded the data? | SINTEF report (23) | Five researchers coded and analyzed the interviews. |
| 25 | Description of the coding tree | Did authors provide a description of the coding tree? | SINTEF report (23) | The full account of the analysis is provided in the SINTEF report (23). A coding tree/system is described there. |
| 26 | Derivation of themes | Were themes identified in advance or derived from the data? | SINTEF report (23) | Themes were derived inductively from the data. |
| 27 | Software | What software, if applicable, was used to manage the data? | SINTEF report (23) | Excel was used as the main software to manage data. |
| 28 | Participant checking | Did participants provide feedback on the findings? | Methods; Results, SINTEF report (23) | Two user representatives were involved throughout the planning and early development phases, including group-based discussions and prototype testing. The exploratory findings themselves were not returned to informants. |
| **Domain 3: Analysis and findings — Reporting** | | | | |
| 29 | Quotations presented | Were participant quotations presented to illustrate the themes/findings? Was each quotation identified? | SINTEF report (23) | Participant quotations are not reproduced in this protocol, which summarizes the exploratory findings as the basis for the requirement specification. Quotations are presented in the SINTEF report (23). |
| 30 | Data and findings consistent | Was there consistency between the data presented and the findings? | Methods, Understanding the Problem; Discussion | Yes. The findings reported in the manuscript follow directly from the exploratory work and are traceable to the SINTEF report (23). |
| 31 | Clarity of major themes | Were major themes clearly presented in the findings? | Methods, Understanding the Problem | Yes. The major findings are presented as a problem statement: the need for systematic assessment of symptoms, late effects and rehabilitation needs; limited awareness and overview of available rehabilitation services; a fragmented system of public and private providers; and the need to reduce inequalities in access. |
| 32 | Clarity of minor themes | Is there a description of diverse cases or discussion of minor themes? | Discussion | Yes. The Discussion addresses challenges that remain insufficiently addressed, including the absence of a national overview of rehabilitation services and possible inequalities in access affecting individuals with low education, limited health literacy, minority backgrounds or disabilities. |

Reference: Tong A, Sainsbury P, Craig J. Consolidated criteria for reporting qualitative research (COREQ): a 32-item checklist for interviews and focus groups. Int J Qual Health Care. 2007;19(6):349-357.
